# Association Between Walking Speed and Mortality in Cardiac Patients with Type 2 Diabetes Involved in a Secondary Prevention Program

**DOI:** 10.3390/jfmk10020181

**Published:** 2025-05-17

**Authors:** Valentina Zerbini, Tommaso Piva, Andrea Raisi, Erica Menegatti, Gianni Mazzoni, Giovanni Grazzi, Simona Mandini

**Affiliations:** 1 Center for Exercise Science and Sport, Department of Neuroscience and Rehabilitation, University of Ferrara, 44121 Ferrara, Italy; valentina.zerbini@unife.it (V.Z.); tommaso.piva@unife.it (T.P.); gianni.mazzoni@unife.it (G.M.); giovanni.grazzi@unife.it (G.G.); simona.mandini@unife.it (S.M.); 2 Department of Environmental and Prevention Sciences, University of Ferrara, 44121 Ferrara, Italy; erica.menegatti@unife.it; 3 Public Health Department, AUSL Ferrara, 44121 Ferrara, Italy; 4 Healthy Living for Pandemic Event Protection (HL-PIVOT) Network, Chicago, IL 60612, USA

**Keywords:** cardiovascular disease, type 2 diabetes, walking speed, cardiac rehabilitation, secondary prevention

## Abstract

**Background**: This study aimed to investigate associations between walking speed (WS) and mortality among cardiac patients with type 2 diabetes. **Methods**: Of the 3328 patients included in the ITER registry between 1998 and 2023, 490 patients diagnosed with type 2 diabetes (mean age 67 ± 9 years) were categorized into tertiles based on WS measured at baseline. Walking speed was measured using the 1 km treadmill walking test (1km-TWT). Cox proportional hazard models were used to examine associations between WS and all-cause and cardiovascular disease mortality, adjusting for demographic and clinical confounders. **Results**: The results showed a significative inverse association between WS and mortality. A total of 205 patients died over a median follow-up of 11 years. Patients with a higher baseline WS reported a lower mortality risk compared to slow walkers. A similar magnitude was confirmed by the sensitivity analysis excluding people who died in the first three years. **Conclusions**: The 1km-TWT is an effective predictor of mortality among cardiac patients with type 2 diabetes and a valuable educational tool for exercise-based interventions in secondary prevention. These findings highlight the efficacy of exercise-based programs to improve physical function and reduce mortality risk, underscoring the importance of promoting exercise as part of long-term cardiovascular disease management.

## 1. Introduction

Cardiovascular disease (CVD) continues to be the leading cause of morbidity and mortality globally, accounting for about 17 million deaths annually [1]. Advancements in intervention strategies and medications have resulted in more people surviving initial events, leading to a greater number of patients requiring long-term cardiovascular risk management [2,3,4]. Among the comorbidities of CVD, type 2 diabetes (T2D) exacerbates health risks with several microvascular complications and is the most prevalent mortality cause [5,6]. Cardiac rehabilitation programs with structured exercise and physical activity emerged as essential tools for secondary prevention with the aim of managing risk factors, improving exercise capacity and enhancing long-term outcomes. Despite guidelines recommending regular daily physical activity, many patients are not regularly active, and almost half of patients with CVD and TD2 do not attempt recommended programs [7].

Walking speed (WS) is a sensitive and reliable indicator of overall health and has been recognized as a vital sign for functional capacity [8,9]. There is strong evidence in support of the relationship between WS and exercise capacity, highlighting the link between physiological responses, health outcomes, and quality of life. Walking speed and exercise capacity are related to factors such as muscle strength, cardiopulmonary function, and neuromuscular coordination, and they can vary depending on factors such as age or sex [10,11].

Consistently, evidence has demonstrated that a faster walking speed is inversely associated with hospitalization or mortality rates when compared with slower walkers, reinforcing the significance of WS as a key health indicator [12,13,14,15]. Despite its potential, few studies have explored the relationship between WS and long-term mortality outcomes in patients with CVS and type 2 diabetes who receive exercise-based interventions [7].

Recent evidence has demonstrated that the average WS maintained during a moderate 1 km treadmill walking test (1km-TWT) was a strong prognostic predictor in patients with CVD [16]. The 1km-TWT also provides a reasonably accurate and simple tool to predict changes in VO2peak due to moderate walking training in outpatients with CVD [17]. Thus, the aim of the current study was to examine the association between WS, assessed through the 1km-TWT, and all-cause and cardiovascular mortality in cardiac patients with type 2 diabetes. By analyzing a large cohort of outpatients over an extended 25-year follow-up period, we aimed to provide evidence supporting exercise-based program integration into secondary prevention strategies.

## 2. Materials and Methods

### 2.1. Subjects and Study Design

This study analyzed data from the ITER study (InTegrating exERcise into lifestyle of cardiac outpatients). ITER is an observational patient registry designed to evaluate the effectiveness of an exercise-based secondary prevention program in individuals with stable cardiovascular disease (ClinicalTrials.gov. NCT05817305, 17 April 2023). The initial cohort included 3328 participants referred from their general practitioner or specialist to the Center for Exercise Science and Sport of the University of Ferrara, Italy, between 1998 and 2023. Patients with T2D and full available data for measured walking speed and covariates were included in the study. Cardiovascular disease history was classified into the following categories: acute myocardial infarction (AMI), percutaneous coronary intervention (PCI), coronary artery bypass graft (CABG), and heart valve repair or replacement. CABG was prioritized over other causes of hospitalization. If AMI was the admitting diagnosis, it was recorded as AMI regardless of whether a subsequent PCI was performed. PCI without a preceding AMI was categorized separately as PCI without AMI. Similarly, valvular replacement in the absence of AMI was coded as valvular replacement. Cases involving cardiac tumors, coronary artery anomalies, or heart transplantation were classified as “other”. Diagnoses were identified based on the International Classification of Diseases, 10th Edition (ICD-10). The study was approved by the Ethics Committee of the University of Ferrara, cod. 105/2023/Oss/UniFe. Written informed consent was obtained from all participants at the time of enrolment.

### 2.2. Measurements

Patients’ follow-up consisted of periodic visits, which included clinical assessments such as medical history, blood chemical analysis, and risk factor monitoring and functional evaluation through the validated 1km-TWT walking test [16,18,19,20,21,22,23,24]. Body mass index (BMI) and blood pressure were measured, and hypertension was defined as systolic blood pressure ≥ 140 mm Hg or diastolic blood pressure ≥ 90 mm Hg [25]. All patients performed the submaximal perceptually regulated 1km-TWT starting at a WS of 2.0 km/h, with a progressive increase of 0.3 km/h every 30 s up to a WS corresponding to a perceived intensity of 11−13/20 on the Borg scale (6–20). The WS was then maintained for 1 km. Speed could be adjusted to maintain a moderate level of perceived effort [18,21]. Time to complete the 1000 m, heart rate, and average and maximum WS were recorded. Polar RS100 was used to monitor heart rate (Polar Electro, Kempele, Finland). Validated short forms of the test over 500 m or 200 m were used with patients unable to walk at WS ≥ 3.0 km/h [26,27,28].

### 2.3. Exercise Intervention and Follow-Up

A team composed by kinesiologists and sport physicians followed the patients during their follow-up, conducting an educational intervention at each visit to reinforce healthy behaviors and encourage physical activity [29]. The patients were instructed to perform home-based exercise, which was tailored based on the results of functional evaluations and continuously individualized. The educational intervention was also carried out during the test, as it allows patients to experience a safe and appropriate walking intensity, inducing a learning effect that influences their unsupervised physical activity [30]. The exercise recommendation was 30 to 60 min of moderate-intensity exercise, to be performed at least 5 days a week, but preferably 7 days a week [31]. Patients were educated to replicate target moderate intensity similar to that kept during the 1km-TWT (i.e., 11–13/20) and to progressively increase duration. All-cause and disease-specific mortality were monitored for all participants. Data on deaths were obtained from the regional Health Service Registry of Emilia-Romagna or through contact with the patient’s general practitioner or relatives. The duration from baseline to death was calculated in months.

### 2.4. Data Analysis

Patients were categorized into three tertiles (Slower, Medium, or Faster) based on their baseline WS, adjusting for age and sex to create homogeneous groups. All-cause and disease-specific mortality were the endpoints for the analysis. Descriptive statistics for continuous variables are presented as mean and standard deviation or as frequency and percentage for categorical variables. Normal distribution was verified through a Kolmogorov–Smirnov test. Statistical comparison of baseline characteristics was performed using a one-way ANOVA for continuous variables and chi-square tests for categorical variables. For each patient, the follow-up ended on the date of death or the end of follow-up (31 December 2023). All the associations were investigated performing Cox proportional hazard models, and all results were reported as hazard ratios (HRs) and 95% confidence intervals (CIs). The Slower group was considered as the reference category. All the associations were adjusted for confounders with three models that included an increasing number of covariates. Model 0 was unadjusted. Model 1 (minimally adjusted model) was adjusted for age, sex, marital status, and education. Model 2 (clinical variables model) was adjusted for all variables in model 1 in addition to myocardial infarction, current smoking, coronary artery bypass graft, family history, hypertension, serum glucose, and dyslipidemia. Schoenfeld residual analysis was performed to assess the assumption of proportionality. The proportional hazard assumption was met by all models. To reduce the potential influence of reverse causality, a sensitivity analysis was conducted, excluding participants who died within the first three years. The level of statistical significance was set at *p* < 0.05. Statistical analyses were performed using R Statistical Software 4.3.2 [R Core Team. A language and environment for statistical computing. Published online 2021. https://www.R-project.org/ (accessed on 20 November 2024)].

## 3. Results

Of the initially enrolled 3328 patients, 470 were initially excluded. These exclusions were based on one or more of the following reasons: inability to complete the test; heart failure classified as class III or higher according to the New York Heart Association [32]; and/or other physical or psychological conditions that interfered with walking ability or missing data for measures or covariates considered in the analysis.

Of the remaining 2858, 2368 did not have a diabetes diagnosis. This resulted in 490 people diagnosed with type 2 diabetes being included in the study. A detailed flowchart of participants included in the analysis is provided in Supplemental Figure S1. The average walking speed measured throughout the test was 3.9 ± 1.2 km/h. The median follow-up period was 11 years (2186.57 person-years, 0.09 average annual death). Demographic and clinical characteristics of patients stratified by tertiles of measured WS are shown in Table 1. Compared to Slower patients, participants in the Faster group were relatively younger. In terms of medical history, they presented a lower percentage of AMI diagnoses but a higher percentage of PCI. Finally, they had a lower overall percentage of medication use.

### Association of Baseline WS with All-Cause and Disease-Specific Mortality

**Figure 1:**
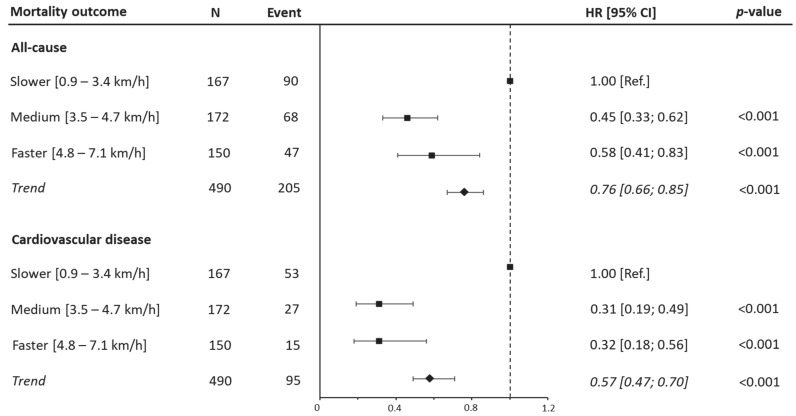
Hazard ratios for all-cause and cardiovascular disease mortality by walking speed categories. Data presented as HRs and their 95% CIs. The reference group was people reporting lower range of walking speed. A hazard ratio for trend was estimated and expressed as risk per one-category increment in walking speed. Analyses were adjusted for age, sex, marital status, current smoking, education, body mass index, myocardial infarction, coronary artery bypass graft, family history, hypertension, and dyslipidemia.

Considering the fully adjusted model, after adjusting for confounders, a decreased risk of all-cause mortality was detected for the medium WS (HR 0.46 [95% CI: 0.33, 0.62 *p* < 0.001]) and faster groups (HR 0.59 [95% CI: 0.41, 0.8 *p* < 0.001]) compared to the slower patients. A similar trend was observed for CVD mortality, with a significant reduction in risk for medium WS (HR 0.31 [95% CI: 0.19, 0.49 *p* < 0.001]) and faster patients (HR 0.31 [95% CI: 0.18, 0.56 *p* < 0.001]) in relation to the reference group (Figure 1 and Supplemental Table S1). Furthermore, considering walking speed as a continuous variable, each unit increment (1 km/h increase) in WS was associated with a 24% reduction in all-cause mortality (HR 0.76 [95% CI: 0.67, 0.86; *p* < 0.001]) and a 42% reduction in CVD mortality (HR 0.58 [95% CI: 0.49, 0.71; *p* < 0.001]). Finally, sensitivity analysis, conducted by excluding the 82 participants who died in the first three years, showed similar results to the main analysis (Supplemental Table S2).

## 4. Discussion

The current study included a cohort of 490 patients with stable CVD and type 2 diabetes encompassing a wide range of age and functional capacity, with the study duration spanning across a total of 25 years of an exercise-based secondary prevention program. The primary finding of this study was the strong inverse association between WS estimated through the 1k-TWT and mortality in patients with stable CVD and type 2 diabetes, reinforcing the role of functional capacity as a key determinant of long-term health outcomes. Consistent with previous research, we highlighted the prognostic value of WS in predicting mortality risk across different populations, including those with CVD and diabetes. The relationship between higher WS and mortality demonstrated a graded reduction in mortality with increasing gait speed [13]. Imran et al. investigated over 21,000 U.S male physicians and found that faster self-reported walking pace was inversely associated with both all-cause mortality and cardiovascular disease. Similarly to our results, they observed a graded reduction in mortality risk with faster walking speeds, independently of exercise frequency and total walking time. However, their population was predominantly healthy older males without specific reference to T2D, while our study specifically targeted a high-risk cohort of cardiac patients with diabetes [33]. In line with our findings, Ueno et al. analyzed over three million subjects and highlighted that faster self-reported gait speed was associated with a lower risk of developing CVD, also finding that this association was amplified in individuals with prediabetes and diabetes. Unlike Ueno et al., who used a subjective single-question gait speed assessment in a younger general population (median age of 44 years), our study employed a standardized 1 km treadmill walk test, providing an objective, reproducible measurement of functional capacity in an older cohort [34].

In addition, previous studies have shown that faster walkers were associated with improved glucose metabolism and a lower risk of developing T2D, further supporting the role of WS as an indicator of cardiometabolic health [35]. The analysis showed a significant 24% reduction in all-cause mortality risk for each 1km/h increase in walking speed. Several mechanisms may explain the protective effect of faster WS on mortality in patients with T2D. Regular physical activity is a well established prevention tool, as it improves insulin sensitivity and glycemic control, thereby mitigating cardiovascular risk factors [36,37]. Moreover, improved functional capacity, as measured by WS, has been linked to better endothelial function and reduced inflammation, which are key mediators of CVD progression [5,35]. However, to the best of our knowledge, the present study was the first to investigate the association between WS and mortality in patients with both stable CVD and T2D with a long follow-up.

From a clinical perspective, these findings underscore the importance of integrating exercise-based interventions into secondary prevention programs for cardiac patients with T2D. Given that the greatest survival benefits were observed over time, structured programs should target progressive increases in gait speed and strategies to promote strong adherence to the programs [7]. Supporting this, previous data from a related cohort within the ITER program demonstrated high long-term adherence rates, with over 75% of the participants achieving the recommended physical activity levels. The same study also showed that WS is a modifiable parameter: participants significantly increased their WS through a structured exercise intervention [29].

Future research should explore whether targeted interventions aimed at increasing WS can directly reduce mortality risk in patients with T2D. Randomized controlled trials assessing the efficacy of different exercise modalities in improving WS and long-term health outcomes are therefore advocated. Additionally, investigating the interaction between WS and other cardiovascular risk factors, such as hypertension and dyslipidemia, may provide further insights into optimizing secondary prevention strategies for individuals with T2D [5].

Finally, these findings further highlight the prognostic value of the 1k-TWT in epidemiological studies and its applicability to real-world clinical settings. Compared to other common walking tests, this test’s moderate intensity and ease of administration enhances its suitability for cardiac patients and older individuals with limited mobility or mild cognitive impairments.

### Strengths and Limitations of the Study

The present research has some strengths, chief among them being the large cohort and the 25-year follow-up period. In addition, it is worth noting that the included data about mortality were accurate, being obtained from regional health records. Finally, the assessment of WS was objective and performed with a validated treadmill test.

However, this study has also several limitations. First, social, behavioral, and psychological factors independently associated with reduced WS were not available. Second, since adherence rates were not determined, establishing a causal relationship between changes in physical activity and WS was not possible. A further limitation of our study is the lack of systematic data on diastolic dysfunction, which is common in individuals with type 2 diabetes and may influence both walking speed and mortality outcomes. Future studies should incorporate echocardiographic parameters of diastolic function to better explore this potential confounding factor.

Moreover, although the flow of participant selection was clearly tracked, we were unable to retrospectively quantify how many exclusions were specifically due to physical limitations such as orthopedic or neurological conditions, as these were not categorized in detail. In addition, although all participants were able to walk independently and complete the treadmill test without assistance, suggesting preserved functional capacity, we did not formally assess frailty status, which may influence both walking speed and mortality risk. Future research should incorporate validated frailty measures to more fully explore this association.

Potential sources of bias should also be considered when interpreting the findings. The study population was composed of individuals who voluntarily enrolled in a secondary prevention program. These patients may represent a more motivated or health-conscious subgroup, potentially limiting the generalizability of the results to the broader population, indicating a need for external validation to generalize the findings beyond motivated subjects. Additionally, despite adjusting for several clinical and demographical variables, the possibility of residual confounding cannot be excluded. Important factors like frailty status, adherence to recommendations and social or psychological variables were not systematically assessed and may have influenced both walking speed and mortality outcomes. Future studies should incorporate these elements and observe associations.

## 5. Conclusions

The average walking speed measured during a moderate and perceptually regulated treadmill walk test effectively predicts mortality among cardiac patients with type 2 diabetes. Our findings reinforce the importance of regular exercise practice promotion as part of secondary prevention programs.

## Figures and Tables

**Table 1:** Baseline characteristics of the 490 patients examined by tertiles of measured walking speed.

Variable	All Patients (n = 490)	Slower (n = 167)	Medium (n = 172)	Faster (n = 150)	*p*-Value
Measured walking speed (km/h)					
Mean (SD)	3.9 (1.2)	2.6 (0.6)	4.1 (0.4)	5.4 (0.5)	<0.0001
Range (min/max)	0.9–7.1	0.9–3.4	3.5–4.7	4.8–7.1	
Demographics					
Age (yr)	67 (9)	69 (9)	64 (10)	63 (11)	<0.01
Sex (women, n, %)	80 (16)	34 (20)	27 (16)	19 (13)	0.2
BMI (Kg/m^2^)	28.7 (4.6)	28.8 (5.1)	28.4 (4.0)	28.9 (4.6)	0.8
LV ejection fraction (%)	58 (8)	57 (8)	58 (9)	58 (11)	0.5
Marital status (married, %)	395 (81)	132 (79)	145 (84)	118 (79)	0.3
Education (high school, %)	206 (42)	71 (43)	70 (41)	64 (43)	0.9
Risk factors					
Family history (n, %)	172 (35)	52 (31)	58 (34)	61 (41)	0.2
Hypertension (n, %)	368 (75)	126 (75)	127 (74)	114 (76)	0.9
Current smoking (n, %)	136 (28)	37 (22)	45 (26)	53 (35)	<0.05
Hemoglobin (mg/dl)	13.5 (1.9)	13.6 (1.9)	13.5 (2.0)	13.4 (1.9)	0.4
Total cholesterol (mg/dl)	180 (47)	187 (51)	176 (47)	178 (42)	0.1
HDL cholesterol (mg/dl)	49 (15)	50 (16)	47 (14)	50 (14)	0.1
Serum triglycerides (mg/dl)	127 (63)	122 (66)	134 (67)	124 (54)	<0.05
Serum creatinine (mg/dl)	1.09 (0.34)	1.08 (0.31)	1.12 (0.37)	1.06 (0.33)	0.1
Serum glucose (mg/dl)	110 (32)	111 (30)	110 (34)	108 (34)	0.059
Medical history					
Myocardial infarction (n, %)	160 (33)	59 (35)	51 (30)	49 (33)	0.5
PCI (n, %)	42 (9)	9 (5)	17 (10)	16 (11)	0.2
CABG (n, %)	223 (46)	72 (43)	83 (48)	68 (45)	0.6
Valvular replacement (n, %)	44 (9)	21 (13)	13 (8)	10 (7)	0.2
Other (n, %)	21 (4)	6 (4)	8 (5)	7 (5)	0.9
Medications					
ACE inhibitor or ARB (n, %)	292 (60)	102 (61)	101 (59)	89 (59)	0.9
Aspirin (n, %)	368 (75)	119 (71)	126 (73)	123 (82)	0.1
β-blocker (n, %)	342 (70)	113 (68)	120 (70)	108 (72)	0.7
Calcium antagonist (n, %)	92 (16)	37 (22)	30 (17)	25 (17)	0.4
Statin (n, %)	317 (65)	100 (60)	105 (61)	112 (75)	<0.05
Diuretic (n, %)	125 (26)	66 (40)	40 (23)	19 (13)	<0.001

Values are presented as mean (SD) or percentage. ARB, angiotensin receptor blocker; BMI, body mass index; CABG, coronary artery bypass graft; HDL, high-density lipoprotein; LV, left ventricular; PCI, percutaneous coronary intervention, stenting, or both.

## Data Availability

The datasets used and/or analyzed during the current study will be available from the corresponding author on reasonable request.

## Supplementary Materials

The following supporting information can be downloaded at: https://www.mdpi.com/article/10.3390/jfmk10020181/s1, Figure S1: Flowchart of participants included in the study; Table S1: Age–sex-specific walking speed combinations and mortality; Table S2: Association between age–sex-specific walking speed tertiles and mortality outcomes (excluding participants who died in the first three years).

## Abbreviations

The following abbreviations are used in this manuscript:

CVD	Cardiovascular disease
WS	Walking speed
T2D	Type 2 diabetes
CRF	Cardiorespiratory fitness
AMI	Acute myocardial infarction
PCI	Percutaneous coronary intervention
CABG	Coronary artery bypass graft
1k-TWT	1 km treadmill walking test
BMI	Body mass index
HR	Hazard ratio
CI	Confidence interval
