# Supplementary material for: Association Between Walking Speed and Mortality in Cardiac Patients with Type 2 Diabetes Involved in a Secondary Prevention Program
Source: J Funct Morphol Kinesiol. 2025 May 17;10(2):181. doi: 10.3390/jfmk10020181 (PMC12101252; doi:10.3390/jfmk10020181)

Journal of Functional Morphology and Kinesiology

Association between walking speed and mortality in cardiac patients with Type 2 Diabetes involved in a secondary prevention program

Valentina Zerbini, Tommaso Piva, Andrea Raisi, Erica Menegatti, Gianni Mazzoni, Giovanni Grazzi, Simona Mandini

Table S1: Age-sex-specific walking speed combinations and mortality

**All-cause mortality**												
	Model 0				*Model 1*				*Model 2*			
	HR	p-value	95% CI		HR	p-value	95% CI		HR	p-value	95% CI	
Slower	1.00	[ref]			1.00	[ref]			1.00	[ref]		
Medium	0.48	<0.001	0.35	0.66	0.46	<0.001	0.34	0.64	0.45	<0.001	0.33	0.62
Faster	0.55	<0.01	0.39	0.79	0.57	<0.01	0.39	0.81	0.58	<0.01	0.41	0.83
**Cardiovascular disease mortality**												
	Model 0				*Model 1*				*Model 2*			
	HR	p-value	95% CI		HR	p-value	95% CI		HR	p-value	95% CI	
Slower	1.00	[ref]			1.00	[ref]			1.00	[ref]		
Medium	0.30	<0.001	0.20	0.51	0.32	<0.001	0.20	0.51	0.31	<0.001	0.19	0.49
Faster	0.31	<0.001	0.17	0.53	0.30	<0.001	0.17	0.53	0.32	<0.001	0.18	0.56

Data presented as HR and their 95% CI. The reference group was people reporting lower range of walking speed. A hazard ratio for trend was estimated and expressed as risk per one category increment in walking speed. Analyses were adjusted for age, sex, marital status, current smoking, education, body mass index, myocardial infarction, coronary artery bypass graft, family history, hypertension, serum glucose and dyslipidaemia.

Table S2: Association between age-sex-specific walking speed tertiles and mortality outcomes conducted by excluding participants died in the first three years.

Mortality outcome	N	Event	HR (95% CI)	*p-value*
**All-cause mortality**				
Slower [0.9 – 3.4 km/h]	137	70	1.00 (Ref.)	
Medium [3.5 – 4.7 km/h]	148	60	0.43 [0.30; 0.61]	<0.0001
Faster [4.8 – 7.1 km/h]	123	34	0.49 [0.33; 0.74]	<0.0001
Trend	*408*	*164*	*0.69 [0.61; 0.80]*	*<0.0001*
**Cardiovascular disease**				
Slower [0.9 – 3.4 km/h]	137	47	1.00 (Ref.)	
Medium [3.5 – 4.7 km/h]	148	24	0.31 [0.19; 0.49]	<0.0001
Faster [4.8 – 7.1 km/h]	123	10	0.32 [0.17; 0.56]	<0.0001
Trend	*408*	*81*	*0.59 [0.49; 0.71]*	*<0.0001*

Data presented as HR and their 95% CI. The reference group was people reporting lower walking speed values. Walking speed was analysed both as a categorical variable and as a continuous variable. A hazard ratio for trend was estimated and expressed as risk per one unit increment in walking speed. Analyses were adjusted for age, sex, marital status, current smoking, education, myocardial infarction, coronary artery bypass graft, family history, hypertension, diabetes, serum glucose, and dyslipidaemia.

Figure S1. Flowchart of participants included in the study

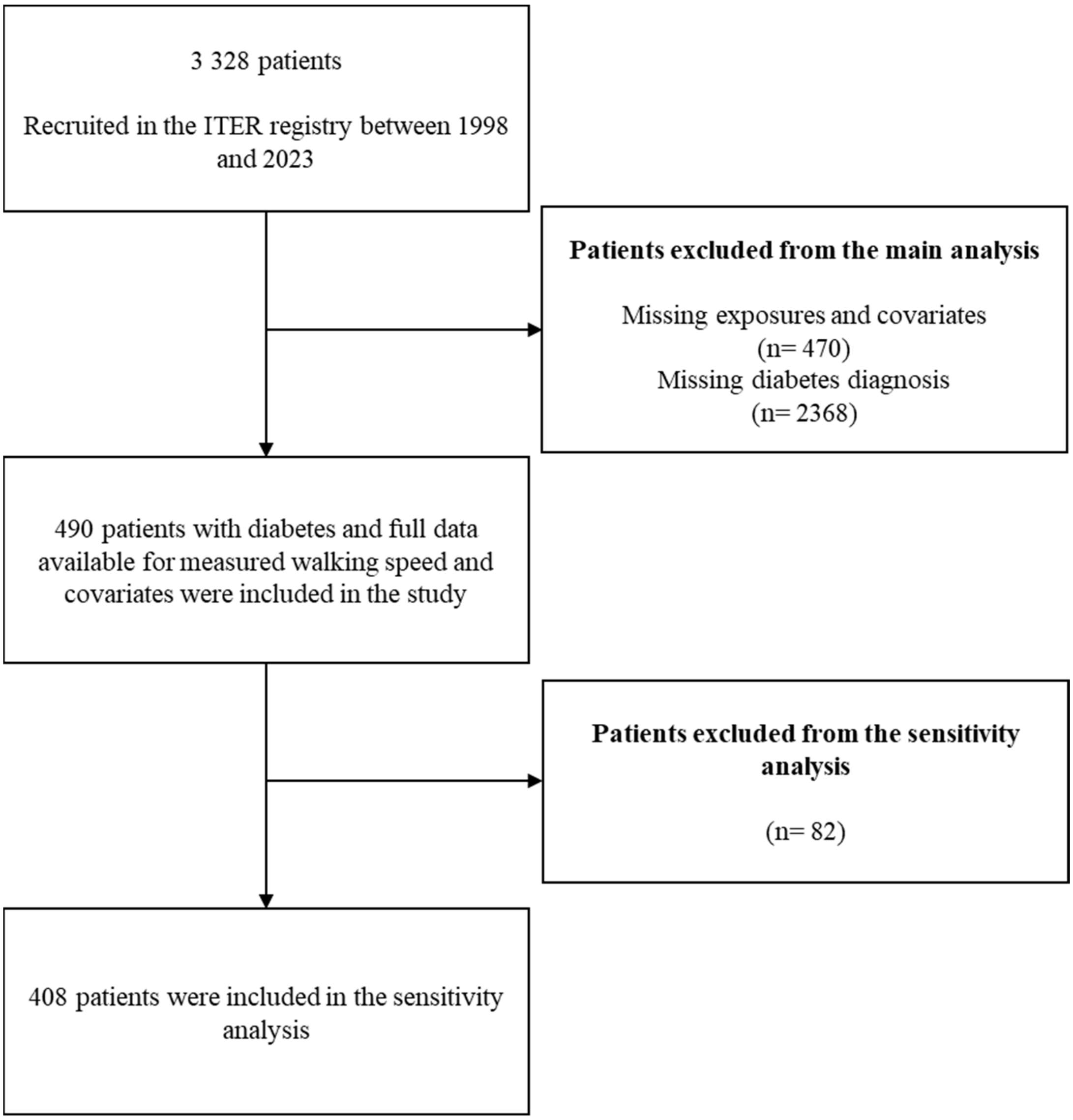

Supplement: Supplementary file 1 [file jfmk-10-00181-s001.zip › jfmk-3511258-supplementary.pdf]
